# The Effect of Alginate Encapsulated Plant-Based Carbohydrate and Protein Supplementation on Recovery and Subsequent Performance in Athletes

**DOI:** 10.3390/nu16030413

**Published:** 2024-01-31

**Authors:** Lotte L. K. Nielsen, Max Norman Tandrup Lambert, Dorte Haubek, Nasser E. Bastani, Bjørn S. Skålhegg, Kristian Overgaard, Jørgen Jensen, Per Bendix Jeppesen

**Affiliations:** 1 Department of Clinical Medicine, Aarhus University Hospital, Aarhus University, Palle Juul-Jensens Boulevard 165, 8200 Aarhus N, Denmark; 2 Municipal Dental Service, Jammerbugt Municipality, Kattedamsvej 34, 9440 Aabybro, Denmark; 3 Department of Nutrition, Division of Molecular Nutrition, Institute of Basic Medical Sciences, University of Oslo, 0317 Oslo, Norway; 4 Department of Public Health, Section of Sport Science, Aarhus University, Dalgas Avenue 4, 8000 Aarhus, Denmark; 5 Department of Physical Performance, Norwegian School of Sports Sciences, 0863 Oslo, Norway

**Keywords:** sports nutrition, nutrition supplement, athletic performance, plant-based, protein, carbohydrate, time-to-exhaustion, alginate encapsulation, hydrogels, oral health

## Abstract

The main purpose of this study was to investigate the effect of a novel alginate-encapsulated carbohydrate–protein (CHO–PRO ratio 2:1) supplement (ALG) on cycling performance. The ALG, designed to control the release of nutrients, was compared to an isocaloric carbohydrate-only control (CON). Alginate encapsulation of CHOs has the potential to reduce the risk of carious lesions. Methods: In a randomised cross-over clinical trial, 14 men completed a preliminary test over 2 experimental days separated by ~6 days. An experimental day consisted of an exercise bout (EX1) of cycling until exhaustion at W_~73%_, followed by 5 h of recovery and a subsequent time-to-exhaustion (TTE) performance test at W_~65%_. Subjects ingested either ALG (0.8 g CHO/kg/hr + 0.4 g PRO/kg/hr) or CON (1.2 g CHO/kg/hr) during the first 2 h of recovery. Results: Participants cycled on average 75.2 ± 5.9 min during EX1. Levels of plasma branched-chain amino acids decreased significantly after EX1, and increased significantly with the intake of ALG during the recovery period. During recovery, a significantly higher plasma insulin and glucose response was observed after intake of CON compared to ALG. Intake of ALG increased plasma glucagon, free fatty acids, and glycerol significantly. No differences were found in the TTE between the supplements (*p* = 0.13) nor in the pH of the subjects’ saliva. Conclusions: During the ALG supplement, plasma amino acids remained elevated during the recovery. Despite the 1/3 less CHO intake with ALG compared to CON, the TTE performance was similar after intake of either supplement.

## 1. Introduction

Endurance sport is often characterised by high training loads and significant metabolic demands [1,2,3], including the depletion of endogenous carbohydrate stores (e.g., liver and muscle glycogen) and damage/recovery cycles of the skeletal muscle proteins [4,5]. Athletes who are engaging in physical exertion, e.g., through competitions or training camps may be required to complete two training sessions a day or on successive days with a limited recovery time in between bouts. Thus, nutritional strategies to optimise recovery have gained a significant amount of attention in order to optimise subsequent performance. It is well established that carbohydrates (CHOs) and lipids are the main fuels oxidised by skeletal muscle during endurance exercise [6,7]. Accordingly, muscle glycogen is a major energy source utilised during endurance exercise, and a direct relationship between fatigue and muscle glycogen levels has been demonstrated [6,7,8], ultimately leading to an impaired performance when glycogen levels become depleted [6,7,8]. The current nutritional recommendations after an exercise bout highlight a high CHO availability with an intake of ~1.2 g CHO/kg/hr during the first hours of recovery [4,9].

In addition, specific amino acids (AAs) and proteins (PROs) may have a potential effect in restoring performance via additional mechanisms that optimise the recovery processes, including the restoration of glycogen stores via insulin-mediated pathways and stimulation of protein synthesis [5,10,11,12,13,14,15]. Previous studies [16,17,18,19] have reported synergistic effects on insulin secretion and protein synthesis to promote muscle damage repair when AAs or proteins are ingested together with CHOs. Moreover, human trials have reported increased protein degradation in skeletal muscle when muscle glycogen stores are reduced or depleted [20,21]. In addition, an early post-exercise intake of proteins has been shown to increase muscle protein synthesis more effectively compared to an intake of energy-equivalent amounts of carbohydrates and lipids only [22]. In line with this, it has been suggested that co-ingestion of carbohydrates and protein may confer an alternate feeding strategy for after endurance exercise to promote recovery and subsequent performance [4,9,19,22,23]. Although questioned, this suggestion finds support from studies where improved recovery and subsequent performance were reported when athletes co-ingested a CHO–PRO supplement compared to CHOs alone [10,23,24,25,26,27,28].

Even though CHOs may be beneficial for performance, excess intake of CHOs within a short period of time can result in inappropriate blood glucose spikes. In addition, endurance athletes have been considered as being at high risk of developing oral diseases, particularly carious lesions, due to the need to consume beverages or gels with a high sugar content during sporting events [29]. A carious lesion occurs as a result of acid formed by bacterial degradation of sugars, and previous studies have assessed the potential risk of carious lesions by measuring the pH of saliva in both diabetic patients and children when consuming high amounts of sugary drinks [30,31,32]. A new alginate-encapsulated CHO supplement (ALG) may present a new solution to optimise performance and recovery, as it contains both protein and CHOs encapsulated in an alginate matrix, which is expected to release CHOs in a controlled manner, targeting the small intestines and releasing less sugar in the oral cavity [33,34]. Alginate is a biopolymer extracted from brown seaweed that is often applied in a wide range of food products, for example as a stabiliser or in the preparation of gel particles, encapsulating micronutrients and probiotics [33,34]. Adding alginate to a CHO beverage has gained attention in the last ~6 years in the field of sports nutrition as a new way to provide CHOs during exercise [35,36,37]. The novelty of ALG is that the CHOs are encapsulated before ingestion, whereas prior studies have added alginate to a beverage, which forms a hydrogel when reaching the low pH level in the stomach [36]. In addition, we have added protein derived from potatoes to the CHO hydrogel, which, to our knowledge, makes this the first study to investigate the effects of encapsulating both CHOs and PRO in alginate hydrogel on performance.

The main purpose of this study was to investigate the effect of the new alginate-encapsulated CHO–PRO hydrogel (ALG) ingested during recovery from prior exercise on subsequent cycling performance. ALG was compared to a commercially available CHO-only supplement (CON), which is not encapsulated in alginate hydrogel. A secondary aim was to assess the effect of the supplements on energy substrates (e.g., glucose, FFAs), hormones (e.g., insulin), and markers of muscle damage (e.g., CK), as well as on the acidity profile of saliva.

## 2. Materials and Methods

### 2.1. Participant Characteristics

Fourteen healthy male participants (mean ± SD; age: 40.4 ± 9.7 years; height: 179.4 ± 7.9 cm; body weight: 81.5 ± 11.5 kg; VO_2_max: 56.8 ± 9.3 mL/min/kg; Wattmax: 397.1 ± 62.7 Watt) volunteered for this study. The subjects were moderate to well-trained endurance cyclists, mountain bikers, or triathletes. The inclusion criteria included male participants aged between 18 and 50 years with a VO_2_max ≥ 50 mL/min/kg. The exclusion criteria included those with metabolic diseases relating to carbohydrate metabolism, and those using medications or dietary supplements or following a low-carbohydrate diet that might affect their carbohydrate metabolism during cycling or recovery. The protocol was approved by the Regional Ethics Committee of Central Denmark (journal no.: 1-10-72-308-18). The study was conducted according to the Declaration of Helsinki and participants gave their informed written consent after an oral briefing and provision of written information about the study (clinical trial no.: NCT05207332).

### 2.2. Experimental Design

**Figure 1:**
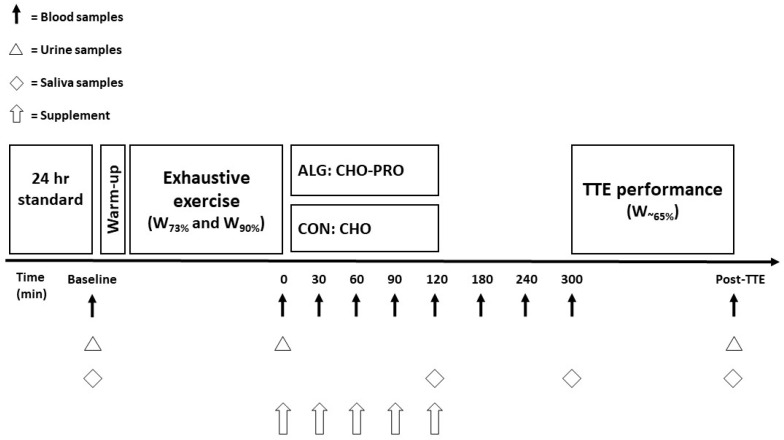
Overview of the experimental design.

This study was conducted as a single-blinded randomised cross-over control study. The participants were blinded to the supplements provided. Subjects completed one session of physiological measures (i.e., a preliminary test), and two experimental interventions separated by approximately six days. Each experimental day consisted of an exhaustive cycling exercise bout (EX1) followed by 5 h of recovery, where the subjects were supplemented with the alginate CHO–PRO (ALG) or a control (CON) supplement in a randomised order. The supplements were given during the first 2 h of recovery. Water was provided ad libitum throughout the study. After 5 h of recovery, the subjects completed a time-to-exhaustion (TTE) performance test. The study’s protocol, including a timeline of its events, is described in Figure 1. Participants consumed a delivered standardised dinner in the evening, which was followed by an overnight fast prior to each experimental day. They were instructed to refrain from heavy and vigorous training on the day prior to the experimental day.

### 2.3. Preliminary Test

Participants met up at the laboratory approximately 6 days before the first experimental day to perform a VO_2_max test on a cycle ergometer (Monark Ergomedic 828 E, Monark Exercise AB, Vansbro, Sweden). Before initiating the test, the participants were introduced to the VO_2_max protocol. They chose their own cadence, which was the range they had to cycle at throughout the whole study (±5 RPM). They started with a warm-up, which was self-chosen from a range of 120–150 watts, for 5 min. Thereafter, the load increased by 30 W every minute until the subjects could not hold the cadence within ±5 RPM. A failure of VO_2_ to increase with the increasing workload as well as a respiratory exchange ratio (RER) > 1.10 was used as the criteria for a successful VO_2_max measurement. The preliminary test served to determine their individual VO_2_max in order to ensure the adherence of the subjects to the inclusion criteria. Wattmax was determined as: workload of the last stage completed + [(30 W/60 s) × seconds at the final stage] [26].

### 2.4. Exhaustive Cycling Exercise (EX1)

On the experimental day, the participants reported to the laboratory in the morning fasted. Baseline plasma, urine, and saliva samples were collected before initiating the first exercise bout (EX1). Water was ingested ad libitum during the exercise bout. The following protocol used in this study was modified from a previous study [24]. The session consisted of ergometer cycling for 3 × 4 min at a workload eliciting ~50%, 55%, and 60% Wattmax, followed by cycling at ~73% Wattmax until exhaustion in 20 min intervals with 5 min recovery between each interval. Exhaustion was defined as the point when subjects were unable to maintain their pre-determined cadence (self-selected cadence ±5 RPM) at their specific workload despite three verbal encouragements. Followed by 5 min of recovery, participants then performed 1 min intervals at ~90% Wattmax followed by a 1 min break. This was repeated until the participants were unable to maintain the required power for 1 min. A previous study [8] has demonstrated very low levels of muscle glycogen when cycling in 20 min intervals until exhaustion. Heart rate (HR) was measured during EX1, including at the approximate mid-point of every sprint, as well as during the TTE performance test (heart rate monitor: Polar H10 heart rate sensor). Throughout the study, the designated recovery periods during exercise protocols were performed as complete rest or under light work (i.e., at ≤100 watts). This was self-selected by the subjects. During the trials, exercise was completed in thermoneutral conditions ~21 °C.

### 2.5. Time-to-Exhaustion Performance Test

Immediately after the first exercise session (EX1), biological samples were collected, and participants were given the first portion of supplement during the first 2 h of recovery. During the recovery period, participants rested on site (Department of Clinical Medicine, Aarhus University Hospital), before initiating the cycling performance test until exhaustion (TTE). Exhaustion was defined as the point in time when subjects were unable to maintain their pre-determined cadence at their specific workload despite three verbal encouragements. The exercise started with a standardised warm-up (see Section 2.4), which was followed by cycling at ~65% Wattmax without stop until voluntary exhaustion.

### 2.6. The Supplements

For the preparation of the alginate gel spherical particles, the extrusion-dripping method was applied [34]. The participants received either CHO–PRO encapsulated in alginate hydrogel (ALG) (Jens Møller Products ApS, Herning, Denmark) or a commercially available CHO supplement (CON), which was not encapsulated in alginate (SIS, Lancashire, UK). ALG contains protein concentrate extracted from potatoes, which contains 53% EAA, 9.8% leucine, 6.3% valine, and 5.4% isoleucine (Protafy 130, KMC, Brande, Denmark). The CHOs in ALG comprise a mixture of glucose and fructose in a ratio of 2:1. The ALG supplement differs from previous studies as the macronutrients are encapsulated in an alginate hydrogel. ALG was provided in the amount of 0.8 g CHO/kg/hr + 0.4 g protein/kg/hr and CON was provided in the amount of 1.2 g CHO/kg/hr. The CHOs in CON were in the form of maltodextrin. The supplements were isocaloric and were supplied in equal portions every 30 min during the first two hours of the recovery period. The supplements were of similar colour and were provided in opaque (dark-coloured) cups. The supplements were provided with water on the side. The order in which the participants received the supplements was randomised simply by drawing lots and was blinded to the subjects only.

### 2.7. Biological Sampling and Analyses

*Blood sampling:* Venous blood samples were taken from a Teflon catheter, which was inserted into an antecubital vein. This was carried out before initiating the EX1 session in order to collect baseline values. Blood samples were taken in 2.0 mL Li-Hep (BD, Plymouth, UK), 1.2 mL APRO-EDTA (SARSTEDT, Nümbrecht, Germany), and 2.0 mL and 4.0 mL EDTA tubes (BD, Plymouth, UK), placed on ice, and centrifuged for 10 min at 4 °C (2500× *g*). Plasma was then pipetted into 2 mL Eppendorf tubes (SARSTEDT, Nümbrecht, Germany) and stored at −80 °C until subsequent analysis. The samples were collected at baseline, immediately after EX1 (i.e., *t =* 0), at *t* = 30, 60, 90, 120, 180, 240, 300 min after EX1 and immediately after the TTE test (i.e., post TTE) (Figure 1). The blood samples were analysed for plasma glucose, insulin, glucagon, free fatty acids (FFAs), glycerol, creatine kinase (CK), and ammonia.

*Glucose:* Plasma glucose content was determined applying an enzymatic reference method GLUC assay (Roche Diagnostics GmbH, Mannheim, Germany) on a Cobas c111 system (Roche, Germany).

*Insulin:* Plasma insulin concentrations were measured with an enzyme-linked immunosorbent assay kit, K6219 (Dako, Glostrup, Denmark).

*Glucagon:* Plasma glucagon concentrations were determined using a radioimmunoassay (RIA) kit (EMD Millipore Corporation, Billerica, MA, USA).

*Free fatty acids (FFAs):* Plasma FFA concentrations were measured with an in vitro enzymatic colourimetric assay for the quantitative determination of non-esterified fatty acids (NEFA-HR) (Wako Chemicals GmbH, Neuss, Germany) using the autoanalyser Cobas C-111 (Roche, Germany).

*Glycerol:* The glycerol content was measured via a direct colourimetric procedure (glycerol Randox, Crumlin, UK) using the autoanalyser Cobas C-111 (Roche, Germany).

*Ammonia (NH3):* The ammonia content was also analysed on the Cobas C-111 analyser (Roche, Germany) using the standard NH3 kit from Roche Diagnostics.

*Creatine kinase (CK):* The CK content was analysed via Siemens Atellica CH 930 (Siemens Healthineers, Erlangen, Germany), using an Atellica CH Creatine Kinase kit (CK_L, ref nr 11097640) (Siemens Healthineers, Erlangen, Germany).

*Amino acids (AAs):* The quantification of plasma AAs was conducted using liquid chromatography–tandem mass spectrometry (LC–MS/MS), which has been described previously [26]. Briefly, labelled isotopes were added to the plasma samples as internal standards; dithioerythritol was then added, resulting in a reduction of disulfides, followed by protein precipitation using 5-sulfosalicyclic acid. Before analysis, the extracts were diluted with an aqueous solution of formic acid [0.5%] and heptafluorobutyric acid (HFBA) [0.3%]. LC–MS/MS was conducted by using a Shimadzu LC-20ADXR Prominence LC system (Kyoto, Japan) coupled to a Sciex QTRAP5500 mass spectrometer with a Turbo V ion source (Framingham, MA, USA). Chromatographic separation was achieved using a Phenomenex Kinetex Core Shell C18 (100 × 4.6 mm, 2.6 μm) LC column (Torrance, CA, USA) with an aqueous solution of acetonitrile gradient mobile phase, formic acid [0.5%], and HFBA acid [0.3%]. For detection, positive-mode multiple-reaction monitoring was used, and for quantification, linear calibration curves of the peak area ratios of analyte and internal standard were used. The coefficients of variation for the analytes were 3.4–6.7%.

*Urine (U):* Samples were collected in urine sample cups at the following time points: baseline, *t* = 0 and post TTE. For each subject, a 12 mL sample was frozen and stored at −80 °C.

*U-carbamide:* U-carbamide was analysed on Siemens Atellica CH 930 using an Atellica CH Uric Acid (UA) kit (ref 11097608) (Siemens Healthineers, Erlangen, Germany).

*U-creatinine:* U-creatinine was analysed on Siemens Atellica CH 930 using an Atellica CH Enzymatic Creatinine_2 (ECre_2) kit (ref 11097533) (Siemens Healthineers, Erlangen, Germany).

*Saliva:* Saliva samples were collected at baseline, 120 min after EX1, 300 min after EX1, and immediately after the TTE test, as shown in Figure 1. The pH value of the saliva samples was measured immediately after collecting the batch by using a pH meter (PHM92, LAB pH METER, Radiometer Denmark A/S, Brønshøj, Denmark). This procedure and method was determined in collaboration with Department of Dentistry and Oral Health, Aarhus University. In addition, this method has been utilised in prior studies assessing the potential risk of carious lesions [31,32].

### 2.8. Statistics

All data were tested for normal distribution with visual inspections (QQ plots) and by using D’Agostino and Pearson omnibus normality tests. Data that were not normally distributed were log-transformed and tested for normality. A two-tailed paired *t*-test was used for area-under-the-curve (AUC) analyses of the plasma glucose, insulin, FFAs, glucagon, glycerol, ammonia, CK, and plasma amino acids. A two-tailed paired *t*-test was also used for data analysis of the saliva samples (at all time points), EX1 exercise bout, TTE performance test, and urine samples, comparing ALG with CON. Data denoted with (¤) were statistically analysed with log data, but shown with raw data. Differences were considered significant when *p* ≤ 0.05. Statistical analyses were performed using GraphPad Prism 10.1.0. All following data are presented as the mean ± SEM, if otherwise not specified.

## 3. Results

### 3.1. Physical Exercise Bouts

*The first exercise bout (EX1):* The workload during EX1 (W_~73%_) was 295 ± 14 watts, and the 1 min sprints (W_~90%_) were performed at 363 ± 17 watts. No differences in time to exhaustion (W_~73%_) or sprint intervals (W_~90%_) were found between the groups (ALG vs. CON, W_~73%_: *p* = 0.36 (¤) and W_~90%_: *p* = 0.85) nor between experimental days (day 1 vs. 2, W_~73%_: *p* = 0.6 (¤) and W_~90%_: *p* = 0.4) (Figure S2, Supplementary Materials), indicating that the subjects were equally depleted on both experimental days. The subjects cycled for 71.9 ± 5.7 min and 78.5 ± 10.7 min during the EX1 bout on experimental days 1 and 2, respectively, and performed 9 ± 1 and 10 ± 1 number of sprints on experimental days 1 and 2, respectively. No differences were found in HRs (beat/min) between ALG and CON during the EX1 bout (*p* = 0.4, Table S1, Supplementary Materials).

**Figure 2:**
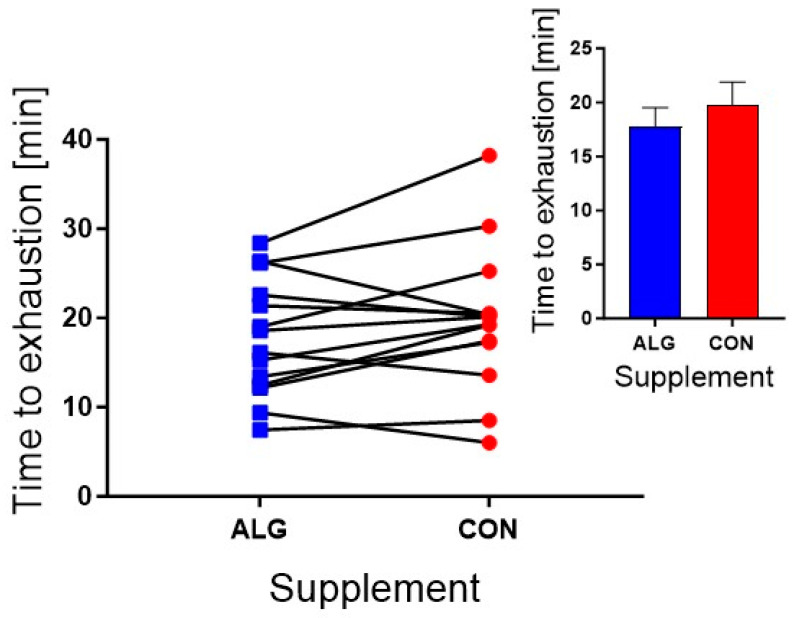
TTE performance test after 5 h of recovery. Data are presented as their mean ± SEM. N = 14 subjects.

*Time-to-exhaustion (TTE) performance test:* The workload used during the TTE test was 262 ± 12 watts. There was no significant difference in the TTE performance test results (*p* = 0.13) between ALG and CON. The average time for TTE performance was 17.76 ± 1.8 min vs. 19.72 ± 2.2 min for ALG vs. CON, respectively (Figure 2). In addition, no differences were found in TTE comparing day 1 vs. day 2 (*p* = 0.23). There was a significant difference in HRs (beat/min) between supplements at the following specific time points: *t* = 4 min, 15 min, and at exhaustion during the TTE test (*p* = 0.03, 168 ± 3 and 161 ± 4 beat/min for ALG vs. CON, respectively; Table S1, Supplementary Materials). At exhaustion, HRs were 172 ± 3 and 165 ± 4 beat/min for ALG vs. CON, respectively (*p* = 0.048) (Table S1, Supplementary Materials).

### 3.2. Supplements and Plasma Analyses

**Figure 3:**
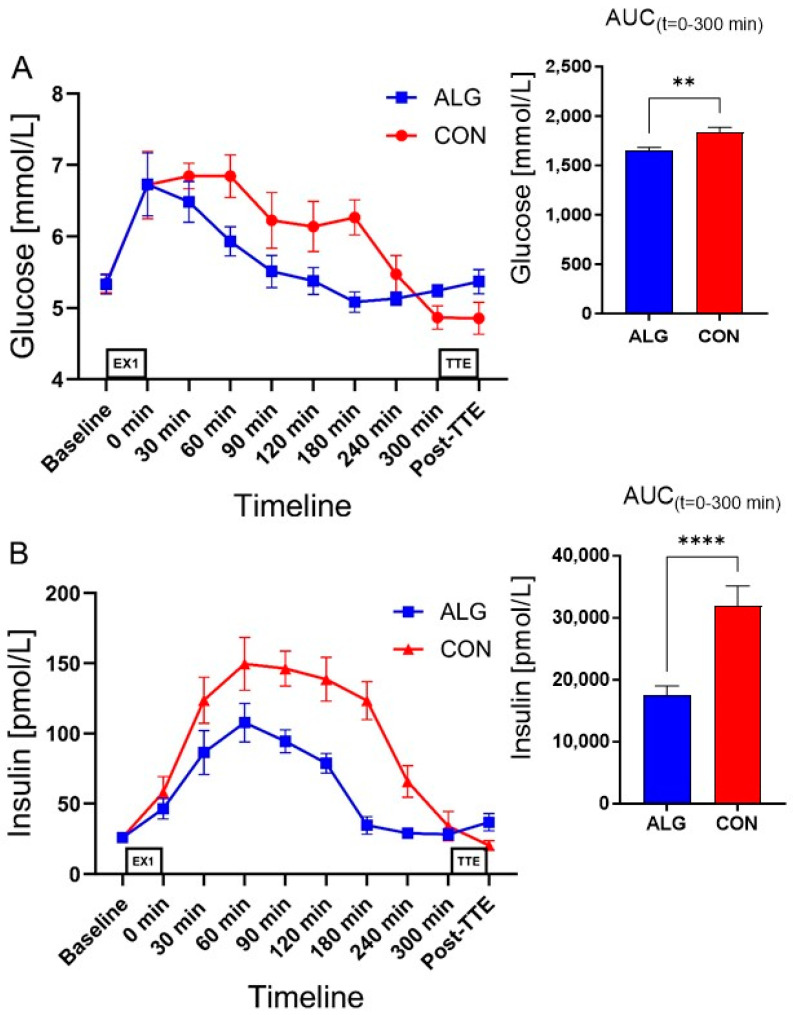

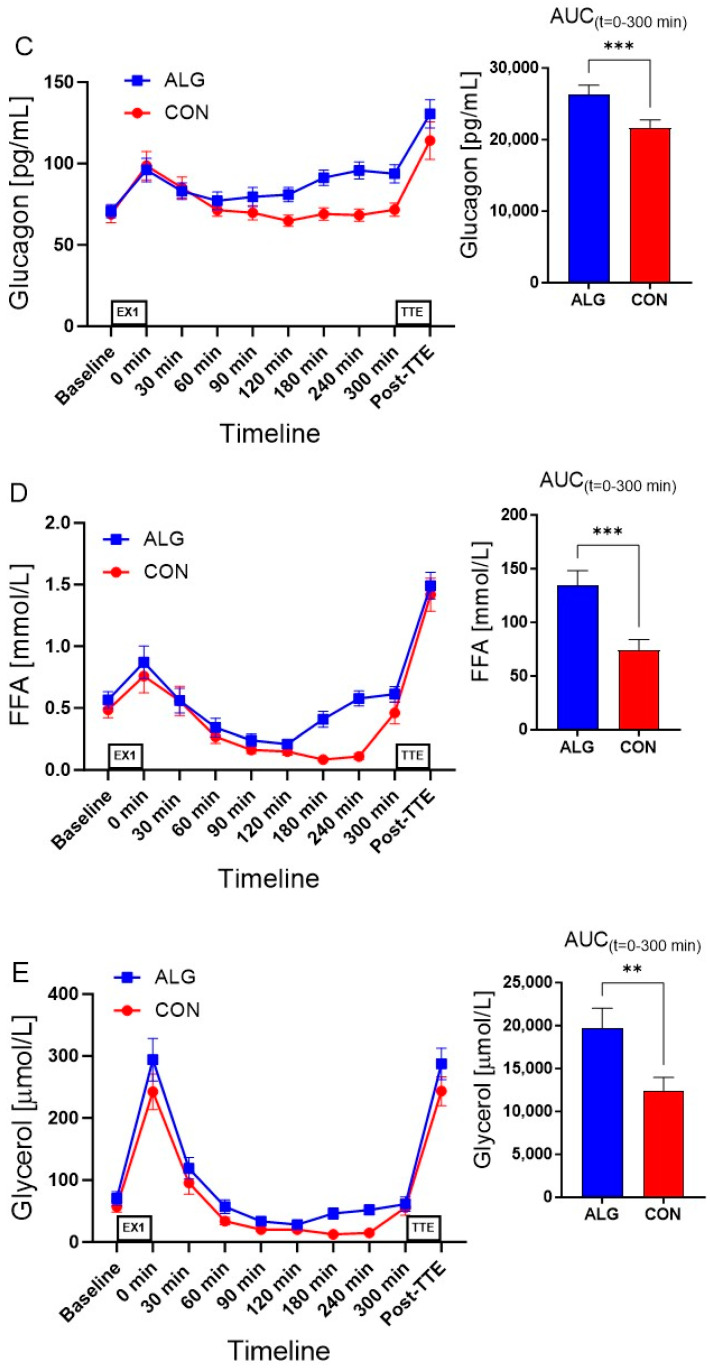

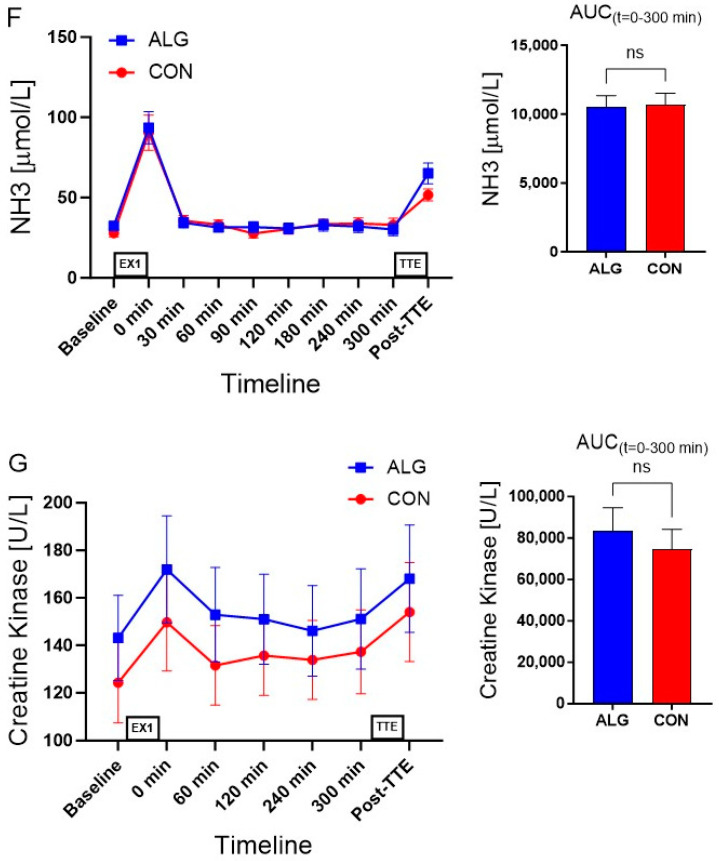
Time-dependent changes in the plasma concentrations of glucose, insulin, glucagon, free fatty acids, glycerol, ammonia, and creatine kinase throughout the clinical trial and area under the curve during the recovery period for ALG and CON. Levels of (**A**) glucose; (**B**) insulin; (**C**) glucagon; (**D**) free fatty acids; (**E**) glycerol; (**F**) ammonia (NH3); and (**G**) creatine kinase are given. Data are presented as their mean ± SEM. ** *p* < 0.01; *** *p* < 0.001; **** *p* < 0.0001; ns: not significant.

Plasma glucose concentrations were significantly lower for ALG vs. CON during the recovery period, i.e., *t* = 0–300 min (AUC: 1651 ± 31 vs. 1828 ± 58 mmol/L for ALG and CON, respectively; *p* = 0.003) (Figure 3). Plasma insulin increased rapidly after intakes of both ALG and CON, but remained lower with ALG vs. CON (AUC: 17,544 ± 1468 vs. 31,894 ± 3231 pmol/L for ALG and CON, respectively; *p* < 0.0001). Plasma glucagon increased during exercise, and decreased rapidly after intake of ALG and CON. Plasma glucagon was higher after intake of ALG vs. CON from 60 to 300 min (AUC: 26,311 ± 1283 vs. 21,599 ± 1153 pg/mL for ALG and CON, respectively; *p* = 0.0002). FFA and glycerol levels increased during exercise and decreased after intake of both ALG and CON. However, the plasma FFA response was significantly higher with ALG vs. CON (AUC: 134 ± 14 vs. 74 ± 10 mmol/L for ALG and CON, respectively; *p* = 0.0002), and plasma glycerol was also higher with ALG vs. CON (AUC: 19,717 ± 2286 vs. 12,435 ± 1525 μmol/L for ALG and CON, respectively; *p* = 0.0018). There were no significant differences between ALG and CON in the plasma ammonia levels, nor were there any significant differences in the plasma creatine kinase (CK) concentrations between the supplements.

### 3.3. Plasma Amino Acids

**Figure 4:**
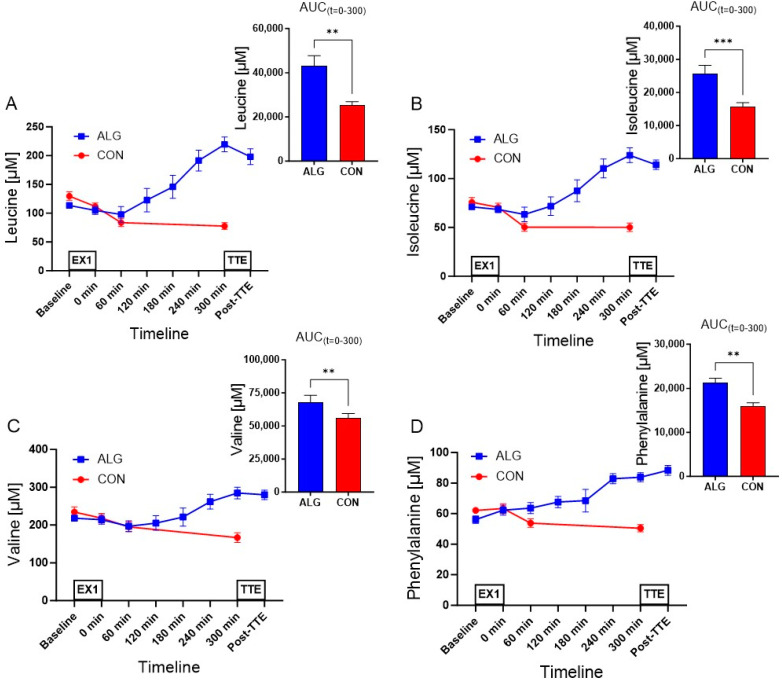

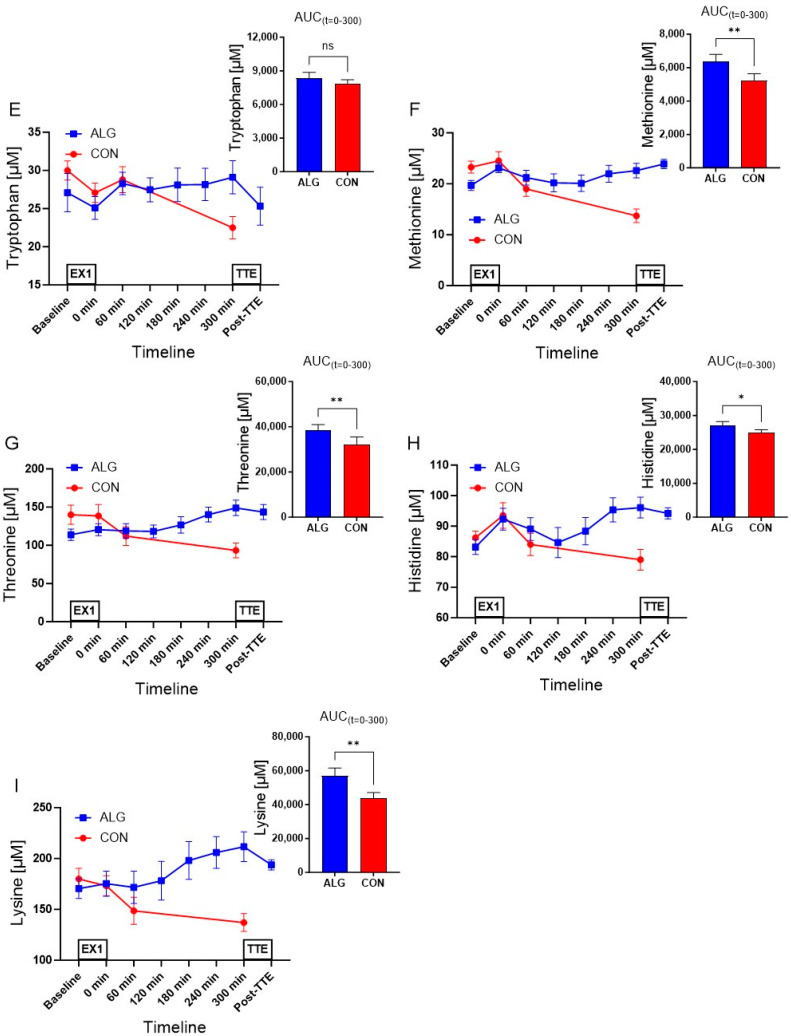
Plasma essential amino acid concentrations during the clinical trial and areas under the curve during the recovery period for ALG and CON supplements. The AAs measured were (**A**) leucine; (**B**) isoleucine; (**C**) valine; (**D**) phenylalanine (**E**); tryptophan; (**F**) methionine; (**G**) threonine; (**H**) histidine; (**I**) lysine. ALG: time points = 8, CON: time points = 4. Data are presented as their mean ± SEM. N = seven subjects. * *p* ≤ 0.05; ** *p* < 0.01; *** *p* < 0.001; ns: not significant.

The levels of plasma branched-chain amino acids (BCAAs) decreased significantly after the first exhaustive exercise bout before the intervention (leucine: *p* = 0.0003, isoleucine: *p* = 0.02, valine: *p* = 0.04, comparing baseline vs. *t* = 0). BCAA levels increased after intake of ALG but decreased with the ingestion of CON. Furthermore, plasma EAA levels increased after intake of ALG compared to CON, and the mean AUC was significantly higher with ALG vs. CON for all essential amino acids, except for tryptophan, although it had a tendency toward statistical significance (*p* = 0.054) (Table 1, Figure 4). With regard to non-essential AAs (NEAAs), the mean AUC was significantly higher after the ingestion of ALG vs. CON for arginine (*p* = 0.006), tyrosine (*p* = 0.005), serine (*p* = 0.009), glutamine (*p* = 0.048), alanine (*p* = 0.02), asparagine (*p* = 0.03), ornithine (*p* = 0.007), and cysteine (*p* = 0.04), but not for the rest (Figure S1, Supplementary Materials).

### 3.4. Urine and Saliva Samples

**Figure 5:**
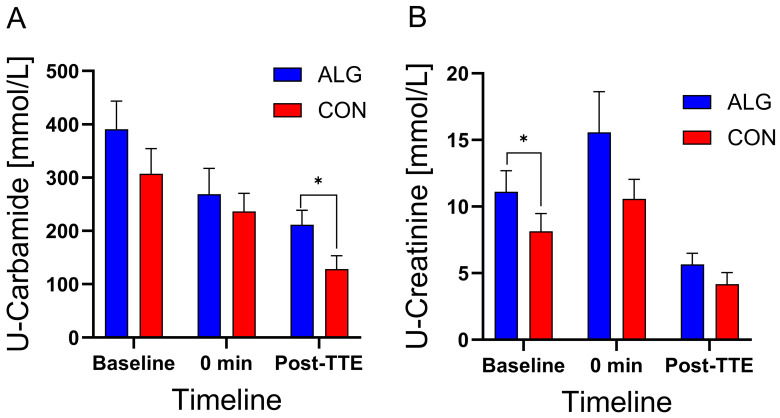
Urine (**A**) carbamide and (**B**) creatinine levels during the clinical trial for ALG vs. CON. Data are presented as their mean ± SEM. *: *p* ≤ 0.05.

**Figure 6:**
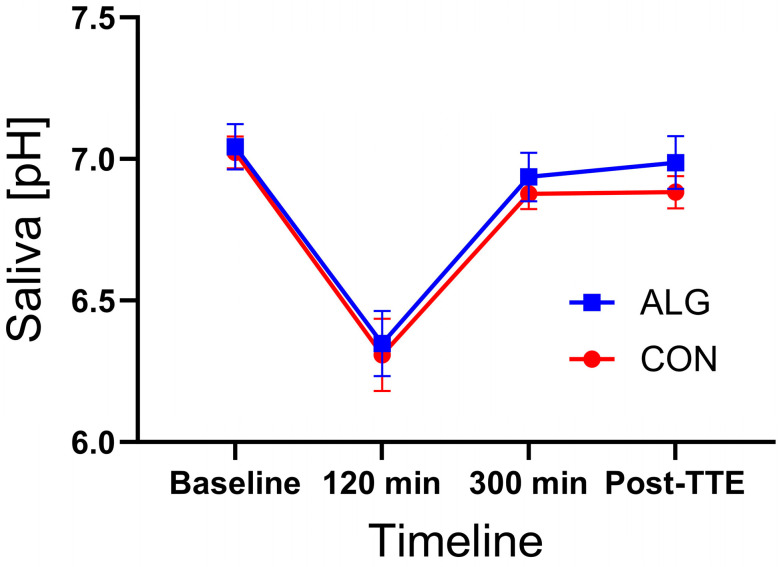
Saliva pH levels during the clinical trial. Data are presented as their mean ± SEM.

U-carbamide concentrations were significantly higher after the TTE performance test when ingesting ALG compared to CON (*p* = 0.013, Figure 5A). There was a tendency for differences to appear at baseline (*p* = 0.053), but no differences at *t* = 0 (*p* = 0.4) appeared between the supplements. In contrast, U-creatinine concentrations were significantly different between supplements at baseline (*p*= 0.02), whereas there were no statistical significant differences at *t* = 0 (*p* = 0.10 (¤)) nor at the post-TTE time point (*p* = 0.13) when comparing ALG with CON (Figure 5B). Moreover, there were no significant differences in the pH values of the subjects’ saliva between ALG and CON (Figure 6).

## 4. Discussion

The primary objective of the present study was to investigate the effect of alginate-encapsulated CHO–PRO supplement (ALG) intake immediately after exhaustive exercise on cycling performance after 5 h of recovery. The novelty of ALG is that the CHOs are encapsulated before ingestion, whereas prior studies have added alginate to a beverage, which forms a hydrogel when reaching the low pH in the stomach [36]. In addition, we have added protein (derived from potatoes) to the CHO hydrogel, which, to our knowledge, makes this the first study to investigate the effect of encapsulating both CHOs and PRO in an alginate hydrogel on TTE performance. Furthermore, we assessed the effect of alginate-encapsulated CHO–PRO on energy substrates (e.g., glucose, FFAs), hormones (e.g., insulin), and markers of muscle damage (e.g., CK). By encapsulating CHO–PRO in alginate, the nutrients are expected to be released in a controlled manner, targeting the small intestines and releasing less sugar in the oral cavity [33,34,35]. To our knowledge, this is the first attempt to encapsulate CHOs and PRO in order to enhance performance and recovery.

Muscle glycogen is a major energy source during endurance exercise and low levels of glycogen have been associated with impaired performance [6,7,8]. In the present study, the intake of CHOs for the CON group was 1.2 g/kg/hr during the first two hours, which is considered an optimal amount for performance [9], whereas in the ALG group, 0.4 g/kg/hr was exchanged with proteins to obtain an isocaloric condition, resulting in an intake of CHOs with ALG of 0.8 g/kg/hr. Despite ALG having 1/3 less CHO intake, the TTE performance was similar between intakes of either supplement. We have previously reported in a similar trial setup, with a 5 h recovery period in between two exhaustive exercise bouts and with similar intakes of CHO–PRO and CHOs only (i.e., CHO: 1.2 g/kg/hr and CHO–PRO: 0.8 g CHO/kg/hr, 0.4 g PRO/kg/hr), that muscle glycogen levels decreased after the first exhaustive exercise bout from ~495 to ~125 mmol/kg dry wt, and that after the recovery period, muscle glycogen increased to similar levels when ingesting CHO–PRO (~311.3 ± 32.8 mmol/kg dry wt) and CHOs (~318 ± 32.1 mmol/kg dry wt) [28]. In the present study, the replacement of CHOs with protein as well as the encapsulation of the nutrients in an alginate matrix did not reduce performance, which suggests that the alginate delivery system may be compatible for recovery.

However, the effect of co-ingesting protein on performance is controversial and many studies show no effect on performance [23]. Some prior studies [10,24,27,28,38] using TTE as the performance test in a laboratory setting have reported a significantly improved performance after co-ingesting CHO–PRO compared to CHOs only. In addition, studies [25,26,39] using a time trial as the performance test have also shown a superior performance after providing CHO–PRO compared to CHOs only. This is not a universal finding [13,40,41,42]. Differences in the protocol design across studies may explain the differences in the performance outcomes when ingestion CHO–PRO, including the depletion protocol prior to the recovery period and the duration of the recovery period. In this study, we found no significant differences in the time to exhaustion in the first exercise bout (EX1) (*p* = 0.6) nor in the number of sprints (*p* = 0.4), indicating that the subjects were equally depleted on both experimental days. However, we did not measure muscle glycogen levels in this study to assess the level of glycogen depletion, and there was inter-participant variation in time in the EX1 bout, where some cycled for under one hour during the depletion ride (Figure S2, Supplementary Materials). Previous studies have supported findings of low levels of glycogen when cycling > 1.5 h [28,43]. Hence, it is possible that not all participants were fully depleted. Former clinical trials [10,23,25,28] implementing a short-term recovery period of 4–6 h have reported an improved cycling performance with CHO–PRO, but this is not universal [13,23,42]. Differences in the amount of calories provided during the recovery period could in part explain the differences in performance outcomes between studies. In line with a prior meta-analysis [23] investigating the effect of CHO–PRO on TTE performance, we found no further effect on TTE performance when the supplements were isocaloric. Matching the supplements regarding their CHO content, thereby ensuring ingestion of CHO in optimal amounts, could be more optimal for future studies investigating the effect of CHO–PRO alginate hydrogels on endurance performance [23,44,45].

In this study, the insulin response was significantly lower when participants ingested the ALG supplement compared to CON during the recovery period. The supplements (ALG and CON) administered were isocaloric, and with the intake of ALG, the subjects ingested 1/3 less CHOs compared to the subjects administered CON due to the addition of proteins, which explains the lower levels of plasma insulin. The combination of CHOs and proteins has been reported to have a synergistic effect in stimulating pancreatic insulin secretion, which, if elevated, may have a potential effect in restoring performance via additional mechanisms that optimise recovery processes, including the restoration of glycogen stores via insulin-mediated pathways as well as stimulating muscle protein synthesis [13,16,17,18,43,46,47]. In line with our findings, a prior investigation by Ferguson-Stegall et al. [25] reported a lower plasma insulin level after the intake of CHO–PRO compared to an isocaloric control (i.e., CHOs only). In contrast, other prior investigations [10,12,26] have shown a higher insulin response with CHO–PRO compared to CHOs only. A potential explanation for this discrepancy could be the type of protein added, as most prior studies used animal protein (whey) rather than protein from a plant-based source (potatoes) as in the present study. The difference in gastric emptying rates of potato protein compared to that of CHOs may have prolonged the intestinal transport of amino acids and CHOs, thus ultimately leading to a lower plasma glucose and insulin response for ALG vs. CON. Interestingly, we found an increased response in FFA, glycerol, and glucagon levels with ALG compared to CON, which could suggest that due to the suboptimal amount of CHOs in ALG in combination with the slowed CHO release from the ALG supplement, it is compensated for metabolically by an increased lipid turnover.

In the present study, we report that AA levels increased and were kept high during recovery after intake of the new ALG supplement. The increase in concentrations of plasma AAs may help prevent the loss of AAs from the muscle, in particular, BCAAs, which have been suggested to be important for protein synthesis and recovery of performance [48]. In addition, the intake of protein may induce a positive protein synthesis after an exhaustive exercise, as exhaustive exercise enhances protein degradation, which continues post exercise [19,20]. We report that the EAAs increased after the ingestion of ALG, containing both CHO–PRO, which was expected [24,26]. The application of alginate encapsulation is utilised in the pharmacy and food industries as a way to protect exposure of functional ingredients such as vitamins and probiotics against the low pH in the stomach [33,34]. This may further ensure enhanced and optimised delivery, e.g., into the duodenum, where the pH is higher. In line with this, implementing alginate encapsulation technology for protein supplementation could be a way to optimise protein utilization via a delayed release in the duodenum [33]. Using the alginate technology for CHOs alone or in combination with other nutrients may also allow for the ingestion of high CHO concentrations without causing gastrointestinal distress [33,34,35]. To our knowledge, this is the first study to investigate the effects of ingesting alginate hydrogel containing both carbohydrates and protein during the early phase of recovery on subsequent TTE performance. Furthermore, this study differs from prior investigations on alginate-CHO supplements as the CHOs were already encapsulated in an alginate hydrogel before ingestion in this study. Prior research provided a CHO beverage with alginate, which forms a hydrogel at pH levels found in the stomach [36,37].

A secondary aim of this study was to assess the acidity profile of saliva. Due to the need to consume beverages or gels containing high amounts of sugars during sporting events, athletes have been considered as being at high risk of developing oral diseases, particularly carious lesions [29,49]. Moreover, previous studies have assessed the potential risk of carious lesions, which occur as a result of acid formed by bacterial degradation of sugars, by measuring the pH of saliva in diabetic patients and children consuming high amounts of sugary drinks [30,31,32]. In this study, no differences were found in saliva pH between supplements and the pH values of saliva were within the normal pH range (pH = 6.2–7.6) for both ALG and CON [32]. There are several factors that may affect dental health, including biological factors (such as the composition of saliva) and behavioural factors (e.g., brushing teeth, eating/drinking habits) [30,31]. More studies are needed to assess the potential risk effects of energy supplements on oral health and related risks, such as the development of carious lesions in endurance athletes, especially during sporting events lasting several days, when the intake of high sugary supplements may be high.

A major strength of the present study is that the subjects were randomly allocated and blinded to the supplementation provided. It had a cross-over design, which minimises the inter-individual differences, as the participants were their own controls. Another strength of this study was that we included 14 participants who were moderate to well-trained cyclists and/or triathletes accustomed to cycling training and/or competitions, thus minimizing the risk of learning effects influencing the performance outcome. Furthermore, a standardised dinner was provided to participants in the evening prior to each experimental day. Standardised isocaloric supplements were provided during the clinical trial. Subjects were asked not to participate in vigorous training or partake in competitions the day prior to the experimental day.

A limitation of this study is that there was no familiarization session. By including a familiarization test, one minimises the test–retest variation. However, the participants were continually active in cycling. Furthermore, in the present study, we did not find any significant differences in time to exhaustion in the first exercise bout (EX1) between experimental days 1 and 2 (*p* = 0.6), nor in TTE performance between day 1 vs. day 2 (*p* = 0.23). During the TTE test, we measured higher HRs at three specific time points, which may suggest that the performance test might have been more stressful with the intake of ALG compared to CON. The participants were encouraged to drink water during the trials, but the absence of accounting for hydration/dehydration differences during the trials is suggested possibly by the heart rate difference. However, it should be noted that only a small number of HR measurements (i.e., at three time points) were included. The present study was not double-blinded and we did not assess GI symptoms. Besides the supplements ingested during the first two hours of recovery, the participants only ingested water. Furthermore, we did not take any muscle biopsies, and we therefore have no data on muscle glycogen levels. Therefore, we cannot be certain in regard to the levels of depletion and repletion. Muscle biopsies were not included in this study’s design due to practical reasons and to ensure the reproducibility of the performance test.

## 5. Conclusions and Perspectives

The main purpose of this study was to investigate the effect of encapsulating CHO–PRO in alginate hydrogel on TTE cycling performance compared to an isocaloric CHO-only supplement (CON). In this study, it was demonstrated that TTE cycling performance was similar after intake of both supplements, despite the 1/3 less CHO content of the ALG compared to CON. The increased glucagon, FFA, and glycerol responses during recovery from exhaustive exercise matched the performance of the CHO control supplement, suggesting that the slowed release and reduced CHO content of ALG was compensated for metabolically by an increased lipid turnover. The intake of ALG containing plant-based proteins increased the EAA levels during recovery. In the present study, we found no differences in saliva pH between the supplements, and the pH-values of the saliva were within the normal pH range (i.e., 6.2–7.6) for both ALG and CON [32]. To our knowledge this is the first study to investigate the effect of CHO–PRO encapsulated in alginate hydrogel. Differences in the protocol design across studies should be considered when interpreting the results of CHO–PRO compared to CHOs only. Moreover, as the CHO content in ALG was suboptimal, it might be difficult to see the additive effect of protein. It would therefore have been interesting to investigate the additive effect of protein by providing an optimal amount of CHOs and matching the supplements for carbohydrate content.

## Figures and Tables

**Table 1:** Mean AUC for plasma Essential Amino Acids.

Variable	ALG	CON	Δ	*p*-Value
*BCAAs* (µM)	62,892 ± 6240	48,693 ± 5788	14,199 ± 1764	<0.0001
*Leucine* (µM)	43,194 ± 4541	25,210 ± 1738	17,984 ± 3025	0.001
*Isoleucine* (µM)	25,756 ± 2398	15,677 ± 1214	10,079 ± 1534	0.0006
*Valine* (µM)	68,077 ± 5173	55,829 ± 3557	12,248 ± 3112	0.008
*Phenylalanine* (µM)	21,341 ± 974	16,032 ± 687	5309 ± 932	0.001
*Tryptophan* (µM)	8348 ± 507	7831 ± 376	517.4 ± 217	0.054
*Methionine* (µM)	6378 ± 404	5230 ± 399	1148 ± 223	0.002
*Threonine* (µM)	38,328 ± 2592	32,169 ± 3318	6158 ± 1062	0.0012
*Histidine* (µM)	27,098 ± 1105	24,887 ± 881	2211 ± 725	0.023
*Lysine* (µM)	56,840 ± 4682	43,940 ± 3127	12,899 ± 2494	0.002

Values are AUC mean ± SEM. Δ is ALG–CON.

## Data Availability

Data are contained within the article and the supplementary material.

## Supplementary Materials

The following supporting information can be downloaded at: https://www.mdpi.com/article/10.3390/nu16030413/s1, Figure S1: Plasma amino acid concentrations during the clinical trial and areas under the curve during the recovery period for ALG and CON products; Figure S2: Time to exhaustion during the exhaustive exercise bout (EXH) before intake of supplements; Table S1: Heart rate response during exercise.
