# Supplementary material for: The Effect of Alginate Encapsulated Plant-Based Carbohydrate and Protein Supplementation on Recovery and Subsequent Performance in Athletes
Source: Nutrients. 2024 Jan 31;16(3):413. doi: 10.3390/nu16030413 (PMC10857232; doi:10.3390/nu16030413)
Supplement: Supplementary file 1 [file nutrients-16-00413-s001.zip › nutrients-2799457-supplementary.pdf]

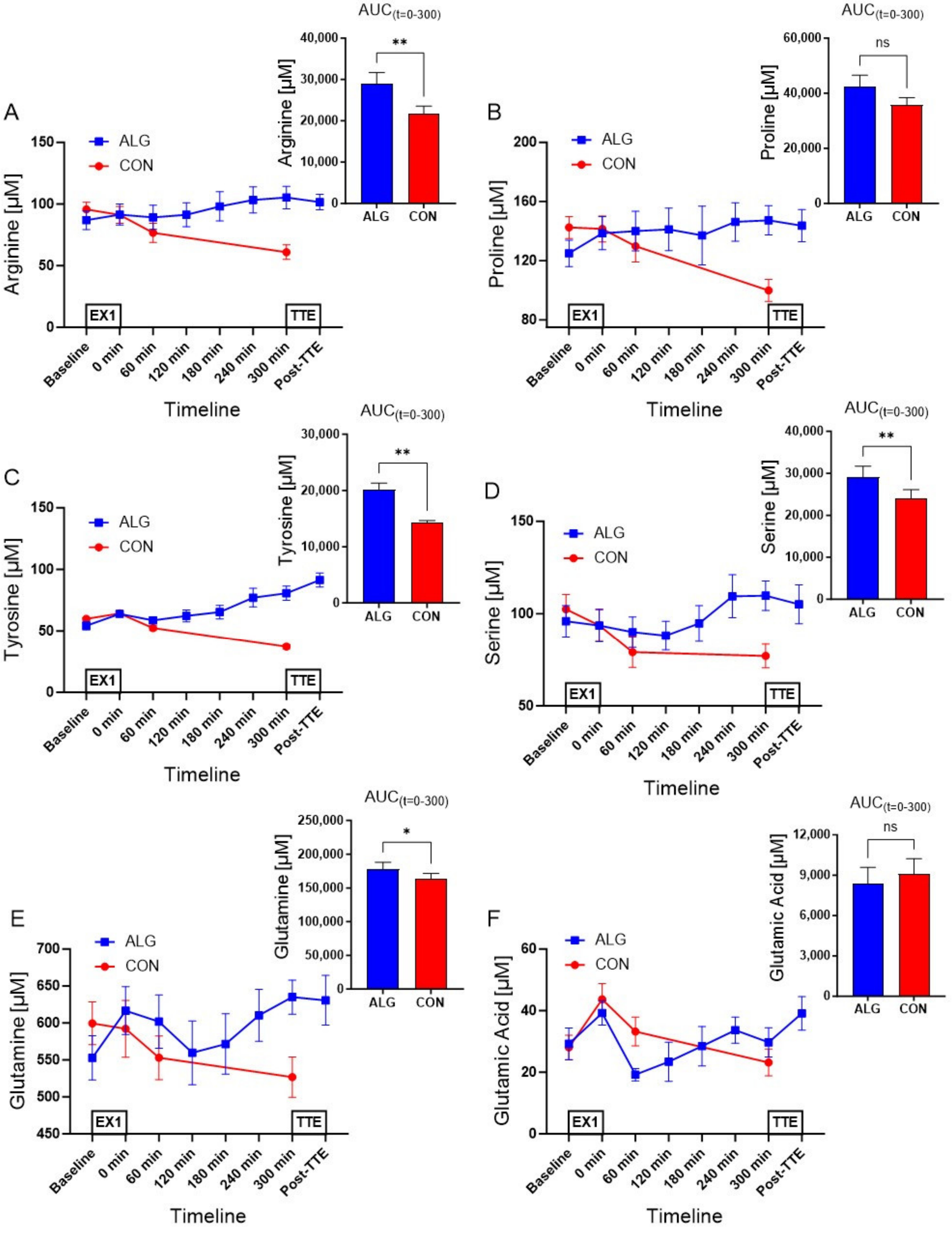
A
Arginine [µM]
ALG
CON
EX1
TTE
Baseline
0 min
60 min
120 min
180 min
240 min
300 min
Post-TTE
Timeline
AUC(t=0-300)
**
B
Proline [µM]
ns
C
Tyrosine [µM]
**
D
Serine [µM]
**
E
Glutamine [µM]
*
F
Glutamic Acid [µM]
ns

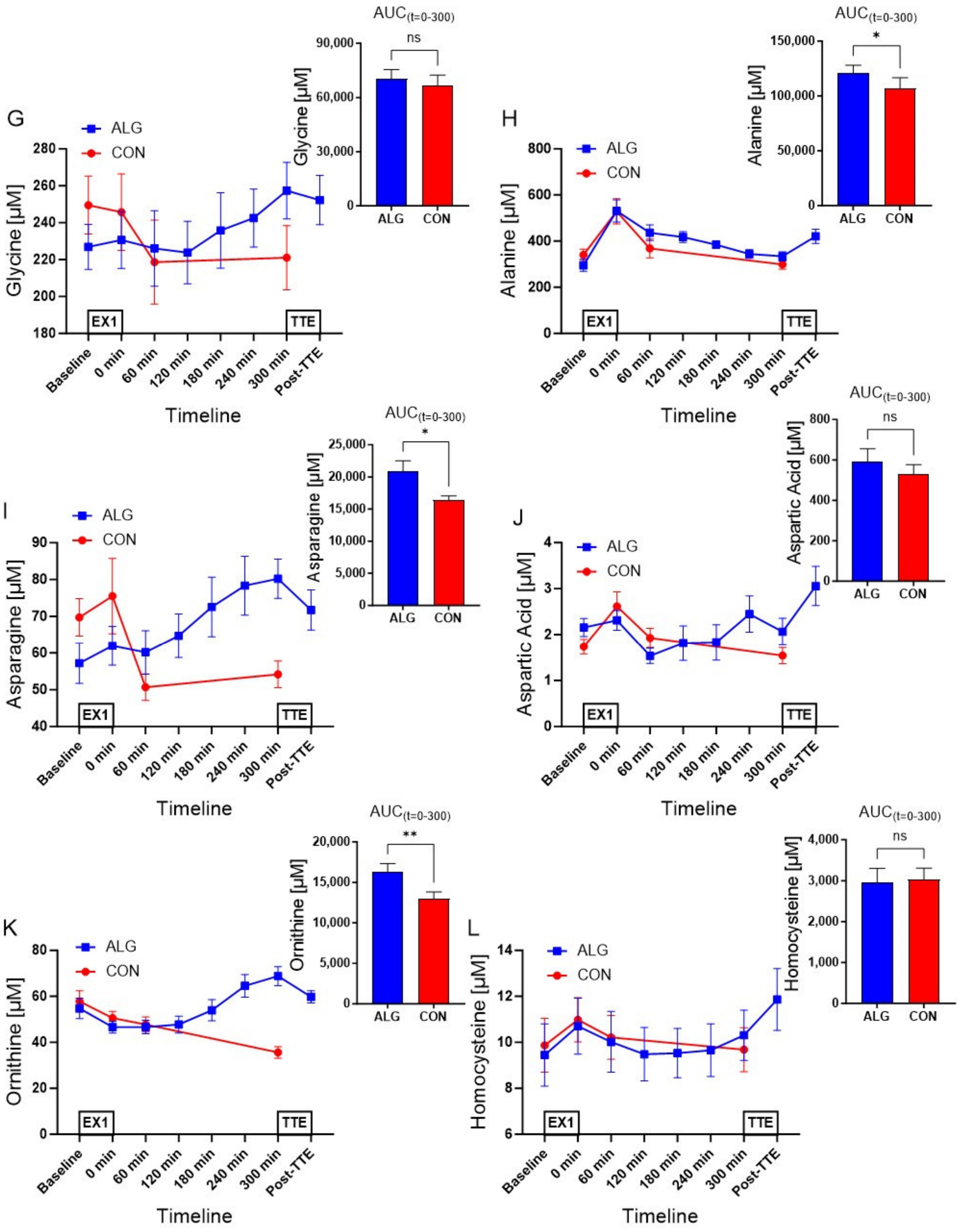

G
ALG
CON
Glycine [µM]
280
260
240
220
200
180
EX1
TTE
Baseline
0 min
60 min
120 min
180 min
240 min
300 min
Post-TTE
Timeline
AUC(t=0-300)
ns
Glycine [µM]
90,000
60,000
30,000
0
ALG
CON
H
ALG
CON
Alanine [µM]
800
600
400
200
0
EX1
TTE
Timeline
AUC(t=0-300)
*
Alanine [µM]
150,000
100,000
50,000
0
ALG
CON
I
ALG
CON
Asparagine [µM]
90
80
70
60
50
40
EX1
TTE
Timeline
AUC(t=0-300)
*
Asparagine [µM]
25,000
20,000
15,000
10,000
5,000
0
ALG
CON
J
ALG
CON
Aspartic Acid [µM]
4
3
2
1
0
EX1
TTE
Timeline
AUC(t=0-300)
ns
Aspartic Acid [µM]
800
600
400
200
0
ALG
CON
K
ALG
CON
Ornithine [µM]
80
60
40
20
0
EX1
TTE
Timeline
AUC(t=0-300)
**
Ornithine [µM]
20,000
15,000
10,000
5,000
0
ALG
CON
L
ALG
CON
Homocysteine [µM]
14
12
10
8
6
EX1
TTE
Timeline
AUC(t=0-300)
ns
Homocysteine [µM]
4,000
3,000
2,000
1,000
0
ALG
CON

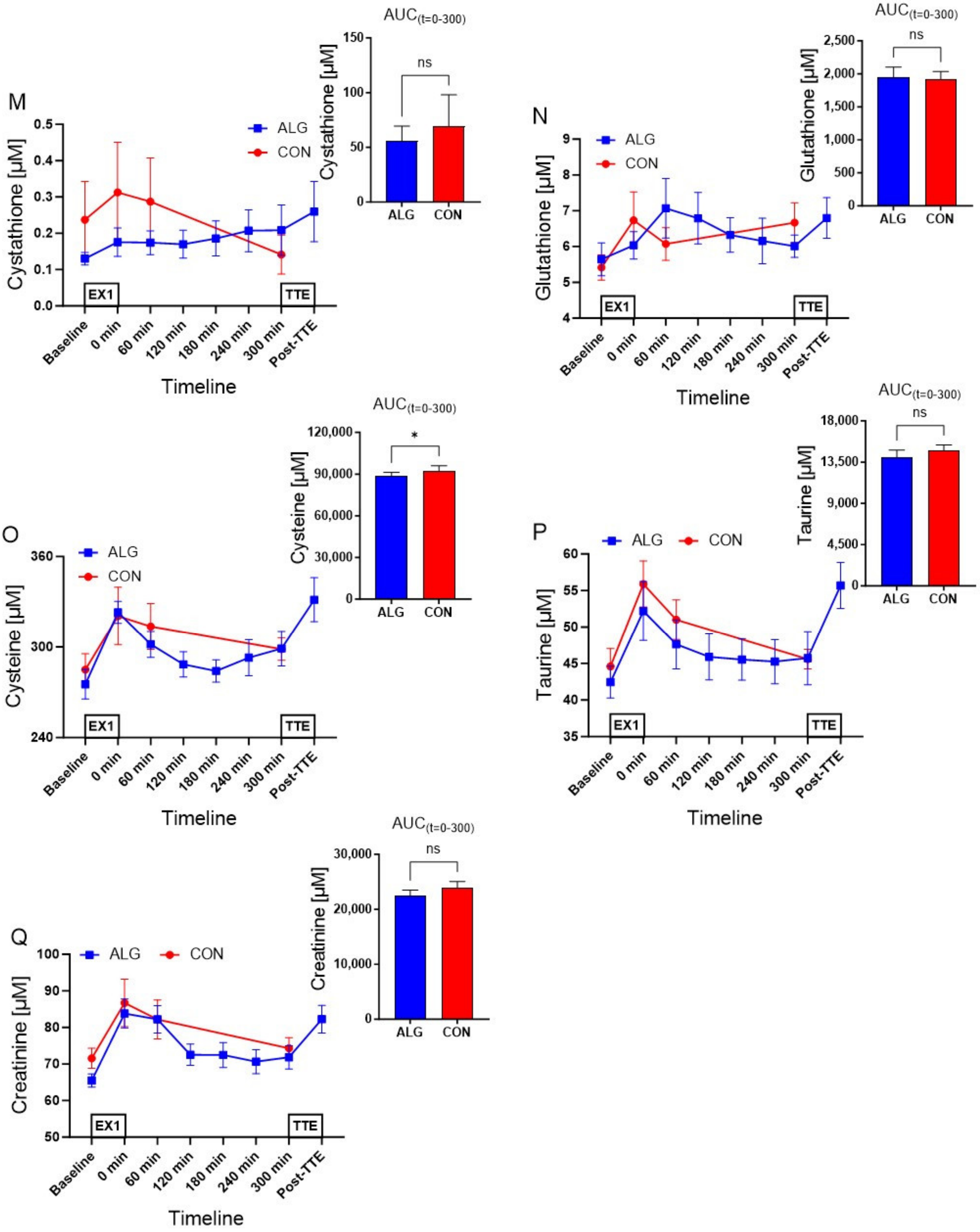
M
Cystathione [μM]
ALG
CON
EX1
TTE
Baseline
0 min
60 min
120 min
180 min
240 min
300 min
Post-TTE
Timeline
AUC(t=0-300)
ns
N
Glutathione [μM]
O
Cysteine [μM]
*
P
Taurine [μM]
Q
Creatinine [μM]

Figure A1. (**A**–**Q**) Plasma amino acid concentrations during the clinical trial and areas under the curve during the recovery period for ALG and CON products. ALG: time points = 8, CON: time points = 4. Data are presented as the mean ± SEM. N = 7 subjects. A data point has been taken out at $t = 0$ for one participant in the plasma aspartic acid response in the CON group as it was 68 standard deviations from the mean. There was no changes in the significance of effect ($p = 0.42$). * $p \leq 0.05$; ** $p < 0.01$; ns: not significant.

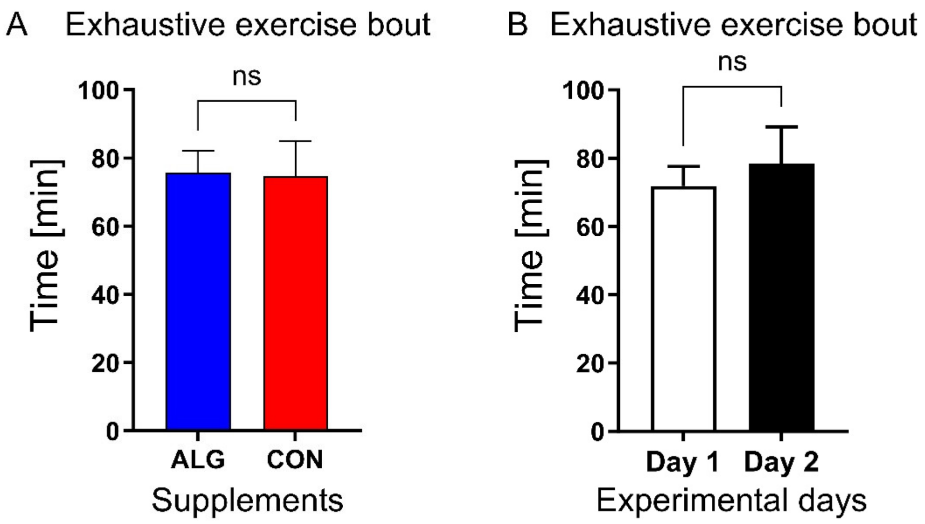

Figure A2. Time to exhaustion during the exhaustive exercise bout (EXH) before intake of supplements. Figure demonstrate time to exhaustion comparison (**A**) between groups (ALG vs. CON) and (**B**) between experimental days (day 1 vs. 2) (¤). N = 14. Data are presented as the mean ± SEM. ns: not significant.

Table A1. Heart rate response during exercise

**(A) Variable**	**Supplement**	**4 min**	**20 min**	**Exhaustion**	**First SP**	**Last SP**
HR–EX1 (beat/min)	ALG	161 ± 4 (14)	171 ± 6 (8)	172 ± 3 (14)	166 ± 4 (14)	172 ± 4 (14)
	CON	163 ± 4 (14)	172 ± 6 (8)	170 ± 3 (14)	165 ± 4 (13)	171 ± 4 (14)
**(B) Variable**	**Supplement**	**4 min**	**15 min**	**Exhaustion**		
HR–TTE (beat/min)	ALG	162 ± 4 (14) *	168 ± 5 (9) *	172 ± 3 (14) *		
	CON	156 ± 4 (14)	163 ± 5 (10)	165 ± 4 (14)		

The heart rate (HR) response during the (A) EX1 bout and (B) TTE test at specific time points. Data are presented as mean ± SEM. The number of subjects at different time points are given in parenthesis; SP: sprint. * Significantly different from CON at the same time point ($p < 0.05$).
